# The Application and Validity of a New Composite Radiographic Index for Patients with Osteonecrosis of the Jaws

**DOI:** 10.3390/diagnostics15070926

**Published:** 2025-04-03

**Authors:** Zafeiroula Yfanti, Sotirios Tetradis, Nikolaos G. Nikitakis, Konstantina Eleni Alexiou, Emmanouil Vardas, Christos Angelopoulos, Kostas Tsiklakis

**Affiliations:** 1 Department of Oral Diagnosis and Radiology, School of Dentistry, National and Kapodistrian University of Athens, 2 Thivon Street, Goudi, 11527 Athens, Greece; kalexiou@dent.uoa.gr (K.E.A.); angelopoulosc@gmail.com (C.A.); ktsiklak@dent.uoa.gr (K.T.); 2 Section of Oral Maxillofacial Radiology, UCLA School of Dentistry, 714 Tiverton, Los Angeles, CA 90095, USA; stetradis@dentistry.ucla.edu; 3 Department of Oral Medicine & Pathology and Hospital Dentistry, School of Dentistry, National and Kapodistrian University of Athens, 2 Thivon Street, Goudi, 11527 Athens, Greece; nnikitakis@dent.uoa.gr (N.G.N.); emvard@dent.uoa.gr (E.V.)

**Keywords:** cone beam computed tomography, medication-related osteonecrosis of the jaws

## Abstract

**Background/Objectives**: This study aims to determine the validity of a recently developed and published index (the modified Composite Radiographic Index—CRIm) as an indicator of disease gravity and progression in the CBCT scans of patients with medication-related osteonecrosis of the jaw (MRONJ) and to detect possible correlations between the radiologic findings and clinical staging of the disease. **Methods**: This study included 43 MRONJ patients with CBCT scans from the School of Dentistry of National and Kapodistrian University of Athens, approved by the Research Ethics Committee. Clinical staging (0–3) was provided based on AAOMS 2022 guidelines. A total of 52 CBCT scans were analyzed, with maxillae and mandibles evaluated separately when both were involved. Two independent observers assessed eight radiologic features, including lytic changes, sclerosis, periosteal reaction, sequestration, non-healing extraction sockets, and other findings (sinus involvement, inferior alveolar canal involvement, and jaw fracture). The CRIm was applied to quantify osseous changes, scoring each feature (0 (absent), 1 (localized/single), 2 (extensive/multiple)), yielding a range of 0–12. For the statistical analysis, Fisher’s exact test and Spearman’s correlation coefficient were used. **Results**: Clinical Stage 1 consisted of 19 jaws, Stage 2 consisted of 16 jaws, and Stage 3 consisted of 17 jaws. No affected jaws were recorded with Stage 0. A statistically significant correlation between the clinical stage and lytic changes, sequestration, and inferior alveolar canal involvement was found (*p*-value < 0.05). Extensive lytic changes, sclerosis, sequestration, periosteal bone formation, and inferior alveolar canal involvement were mostly observed in clinical Stage 3. Furthermore, a statistically significant correlation between clinical stage and CRIm classification was found (rho = 0.446; *p*-value < 0.001). **Conclusions**: The CRIm tends to increase as the clinical stages of MRONJ advance, suggesting a correlation between them.

## 1. Introduction

Medication-related osteonecrosis of the jaw (MRONJ) is a rare but serious complication that must be differentiated from other forms of osteonecrosis of the jaw (ONJ) through detailed clinical history and examination. It is primarily associated with medications such as bisphosphonates, denosumab, and (potentially) antiangiogenic agents [1].

A recent multicenter study examining the characteristics, risk factors, and management strategies for MRONJ found that among patients with osteoporosis-related MRONJ, the most commonly used medications were denosumab (37%), alendronate (26%), and ibandronate (19%). In patients with metastatic bone disease-related MRONJ, denosumab (42%) and zoledronic acid (39%) were the most frequently prescribed treatments [2].

The American Association of Oral and Maxillofacial Surgeons (AAOMS) states that “The case definition of MRONJ includes all the following elements: current or previous treatment with antiresorptive therapy alone or in combination with immune modulators or antiangiogenic medications, exposed bone or bone that can be probed through an intraoral or extraoral fistula(e) in the maxillofacial region that has persisted for more than 8 weeks and no history of radiation therapy to the jaws or metastatic disease to the jaws” [1].

In 2007, the AAOMS introduced a staging system for MRONJ, which was subsequently refined in a 2022 position paper [1,3,4]. The current classification system consists of the following stages: “Stage 0”—patients with no clinical evidence of necrotic bone but who present with nonspecific symptoms or clinical and radiographic findings; “Stage 1”—exposed and necrotic bone or fistula that probes to the bone in patients who are asymptomatic and have no evidence of infection/inflammation; “Stage 2”—exposed and necrotic bone or fistula that probes to the bone, with evidence of infection/inflammation; and “Stage 3”—exposed necrotic bone extending beyond the region of the alveolar bone, pathologic fracture, extraoral fistula, or oral antral/oral-nasal communication [1].

Although there is consensus on the importance of diagnostic imaging in evaluating the extent of MRONJ, a possible link between the radiologic findings (kind, extent, etc.) in MRONJ patients and their contribution to clinical staging remains unclear [5,6]. Radiologic findings for MRONJ may include lytic changes, sclerosis, sequestrations, and periosteal bone formation. None of these findings are specific and can be seen in other conditions such as osteoradionecrosis, osteomyelitis, and jaw metastatic disease [7,8,9].

Clinically, jaw osteonecrosis manifests symptoms such as pain, edema, fistula(e), and bone exposure. Radiographically, it can present a combination of osteosclerosis, osteolysis, and bone sequestration. Although imaging is not currently integrated into the staging of these diseases, it remains essential for assessing disease extent and localization, surgical planning, and postoperative monitoring [8]. Notably, bone necrosis may develop before clinical symptoms appear, highlighting the potential role of imaging in early detection and diagnosis [7].

In this context, a new, modified Composite Radiographic Index (CRIm) was introduced in a study by Yfanti Z. et al. [9] to evaluate the presence and extent of radiologic findings for medication-related osteonecrosis of the jaws, as well as other pathological entities with similar radiologic appearance, such as osteoradionecrosis, osteomyelitis, and jaw metastatic disease, via cumulative radiologic features.

**Figure 1:**
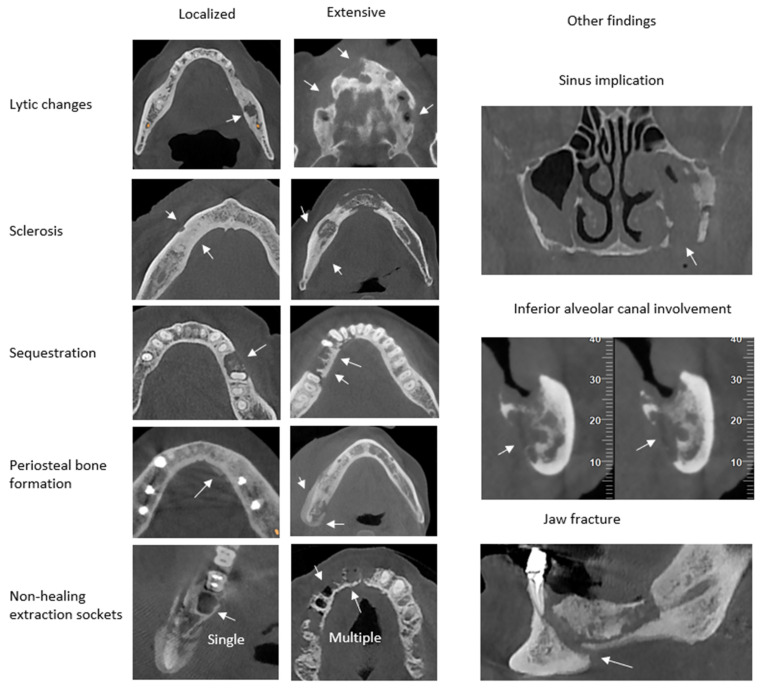
Examples of CRIm scoring for severity of radiologic findings in CBCT images.

The CRIm is based on the Composite Radiographic Index (CRI) introduced by Walton K [7]. The CRI evaluates four radiologic parameters: lytic changes, sclerosis, periosteal bone formation, and sequestration. To improve the existing CRI, two parameters common in the above-mentioned diseases were added to the CRIm. This addition resulted in eight radiologic features that can be classified and evaluated: lytic changes, sclerosis, periosteal bone formation, sequestration, non-healing extraction sockets, and other findings. Other findings include maxillary sinus implication, inferior alveolar canal involvement, and jaw fracture [9]. Lytic changes, sclerosis, periosteal bone formation, and sequestration are scored as follows: (0) for absence, (1) for localized lesions extending up to 1 cm, and (2) for extensive lesions extending 1 cm or multiple lesions. Extraction sockets showing no sign of healing are evaluated only if they are near the lesion, and are scored as follows: (0) absent, if no sockets are present; (1) localized, for the presence of one socket; and (2) extensive, for the presence of multiple sockets. The other findings are individually scored as (0) when absent and (1) when present [9] (Figure 1).

This study aims to determine the validity of the CRIm as an indicator of disease gravity and progression in CBCT scans of patients with medication-related osteonecrosis of the jaw (MRONJ). Additionally, we aim to detect possible correlations between the radiologic findings and the clinical staging of MRONJ, ensuring consistency with the CRIm development established in our previous study, in which the index was introduced [9].

## 2. Materials and Methods

This retrospective study included 43 patients from the institutional database of the National and Kapodistrian University of Athens (NKUA), School of Dentistry, between 2019 and 2023. It was approved by the Research Ethics Committee of the NKUA, School of Dentistry (protocol number 469/29.06.2021). An informed consent form was signed by all patients. Clinical staging for each patient (Stages 0–3) was provided by the Department of Oral Pathology and Hospital Dentistry according to AAOMS 2022 guidelines [1].

Inclusion criteria comprised patients with a definite diagnosis of MRONJ who were referred for a CBCT examination as part of their clinical assessments. All primary disease characteristics and treatment histories of the patients were included in this study. The collected patient data included the following: age, gender, underlying primary disease, date of initial consultation, antiresorptive medication, and location and dimensions of the clinically visible exposed bone. No age or gender restrictions were applied.

Exclusion criteria were applied to patients with a history of head or neck radiation. Furthermore, follow-up scans of the same patients were excluded, except in cases where documented disease progression with additional radiologic findings was observed. Last, in patients with both jaws affected, each jaw was evaluated separately.

As a result, 52 CBCT scans of MRONJ cases matched the inclusion criteria and were included and evaluated. The CBCT scans were performed with the NewTom VGi CBCT scanner (Cefla, Imola, Italy) and were all of acceptable diagnostic quality. The exposure settings were 110 kV and 3–5 mA with 3.6 sec. exposure time. Several large fields of view were used with high-resolution reconstructions (0.125–0.300 mm voxel size).

The CBCT scans under evaluation were anonymized, and two observers (a PhD Candidate in oral and maxillofacial radiology (Z.Y.) and a senior oral and maxillofacial radiologist (K.E.A.)) independently evaluated them under identical standardized viewing conditions. The observers were given specific verbal and written instructions regarding the upcoming CBCT evaluation and were trained and calibrated prior to the evaluation in three separate training sessions. The training sessions included a joint assessment of 20 CBCT scans and the data collection process (radiologic findings collection and recording).

The detected lesions were localized into the following categories: maxillary anterior, maxillary posterior, mandibular anterior, and mandibular posterior sextants. The anterior sextants of both the maxilla and the mandible were defined as the regions within the canines. Lesions extending across multiple sextants were recorded in the respective affected sextants.

Each observer was provided an answer form for each CBCT scan, where they recorded the localization, pertinent radiologic features of pathological findings, and the final CRIm score [9]. The CRIm was determined by summing up the individual scores for each radiologic feature for every jaw examined, yielding a value between 0 and 12. All CBCT scans were classified into three groups according to their CRIm scores: low group (0–3), medium group (4–8), and high group (9–12). In cases with a difference of more than 1 in the CRIm between the two observers, both observers re-evaluated the CBCT scans to achieve consensus. If there was a disagreement between the two observers, a third (K.T., an experienced specialist in oral and maxillofacial radiology) assessed the CBCT scans under identical viewing conditions.

To evaluate intra-rater variability, the observers were asked to independently re-assess a subset of 18 CBCT scans randomly selected from the main dataset. The re-evaluation was conducted after a “washout” period of 4 weeks.

The data were analyzed with descriptive statistics using frequency, incidence rate, mean values, and standard deviation (SD). All statistical analyses were performed using IBM SPSS version 25 (SPSS Inc., 2003, Chicago, IL, USA). Fisher’s exact test and Spearman’s correlation coefficients were applied to correlate both the radiologic features and CRIm with the clinical stage. *p*-values < 0.05 were considered statistically significant. Kappa statistics was performed to evaluate the agreement between the two observers. The intraclass correlation coefficient (ICC) was applied to assess the intra-observer agreement.

## 3. Results

This study included 43 patients (17 males and 26 females) with an average age of 65.4 years and a standard deviation (SD) of 11.2 years. Age ranged from 39 to 87. Out of the 43 patients, 52 CBCT scans with radiologic findings were included and evaluated accordingly. The agreement rate between the two observers was recorded as excellent both for localization (Kappa 0.944-1) and for the presence of radiologic features (Kappa 0.961-1). The intraclass correlation coefficient ranged from 0.94 to 1 for both observers regarding localization and from 0.94 to 1 and 0.89 to 1 regarding the presence of radiologic features for Observer 1 and Observer 2, respectively.

Table 1 presents the localization of MRONJ in the maxillae and mandibles divided into sextants (13 maxillae and 39 mandibles). The most common localization was in the posterior sextants of the mandible (63.5%), followed by the anterior sextants of the mandible (34.6%), the posterior sextants of the maxilla (21.2%), and the anterior sextants of the maxilla (13.5%).

**Figure 2:**
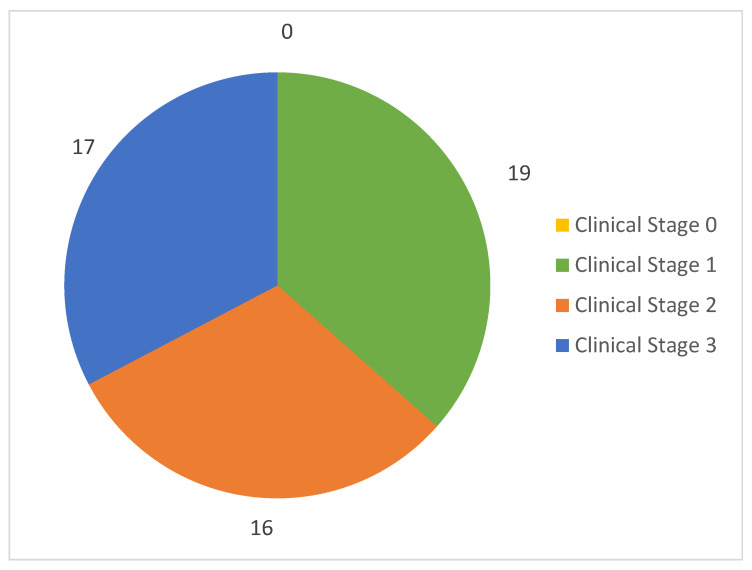
Distribution of the 52 CBCT scans with MRONJ findings according to the clinical stage.

Figure 2 presents the distribution of the 52 CBCT scans with the MRONJ findings according to the clinical stage of the disease. No affected jaws were recorded for Stage 0. Clinical Stage 1 consisted of 19 jaws (36.5% of the sample), Stage 2 consisted of 16 jaws (30.8%), and Stage 3 consisted of 17 jaws (32.7%).

Table 2 demonstrates the correlation between the clinical stage of MRONJ and the various radiologic features. A statistically significant correlation was observed between the “clinical stage” and lytic changes, sequestration, and inferior alveolar canal involvement (*p*-value < 0.05). Extensive lytic changes, sclerosis, sequestration, and periosteal bone formation were mostly observed in clinical Stage 3. Furthermore, inferior alveolar canal involvement was mainly observed in clinical Stage 3.

**Figure 3:**
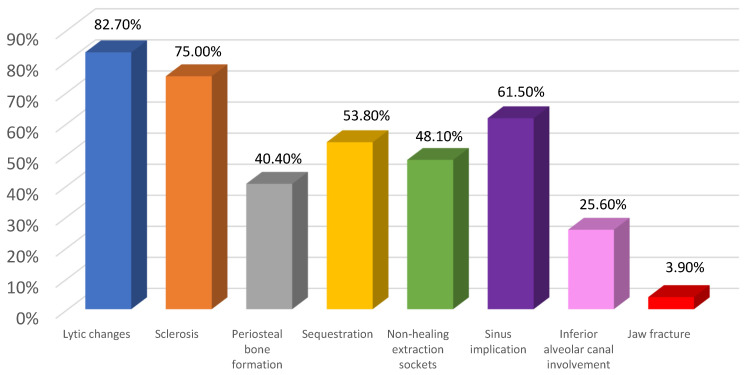
Distribution of the various radiologic features of MRONJ across all patients/jaws.

Figure 3 presents the distribution of all the radiologic features for MRONJ-affected jaws.

Table 3 demonstrates the correlation results for the clinical stage and the CRIm. In clinical Stage 1, approximately half of the affected jaws (47.4%) were classified in the low CRIm group, 75% of the clinical Stage 2 cases were classified in the medium CRIm group, and 41.2% of the clinical Stage 3 cases were classified in the high CRIm group. In addition, a statistically significant but moderate correlation between the clinical stage and the CRIm classification was observed (rho = 0.446; *p*-value < 0.001).

**Figure 4:**
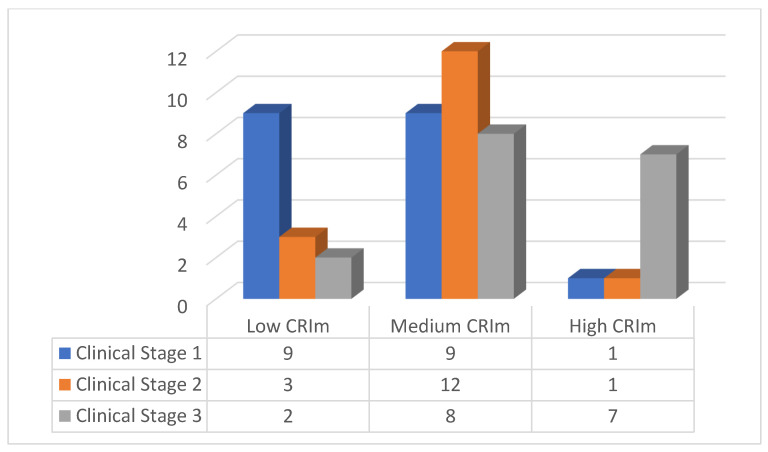
Sample distribution according to CRIm classification in three groups for each clinical stage.

Figure 4 shows the distribution of the CRIm classifications in three groups, according to the clinical stage.

## 4. Discussion

Medication-related osteonecrosis of the jaw (MRONJ) is a rare but serious complication in patients treated for bone malignancies or osteoporosis [10,11]. The AAOMS staging system classifies MRONJ mostly based on clinical signs and symptoms, but radiological imaging offers a more precise assessment of the condition’s extent [1,12,13]. The modified Composite Radiographic Index (CRIm), introduced by Yfanti et al. in 2023 [9], is an updated version of the CRI index from Walton et al. (2019) [7]. It was designed to evaluate the radiographic findings in patients with MRONJ and detect possible correlations between the clinical stage and severity of radiologic changes. The CRIm classifies the severity of radiographic findings into three categories: low (0–3), medium (4–8), and high (9–12).

In the present study, the majority of patients were women (60%), agreeing with findings from similar studies [9,14,15]. This is probably due to the higher prevalence of osteoporosis treatments among female patients.

In our study, 66% of the MRONJ cases affected the mandible, agreeing with the literature [16]. Interestingly, 12% of the patients in our study presented with MRONJ both in the maxilla and mandible, which is considered uncommon [7,17].

We decided to include CBCT as the imaging method to assess the extent of osseous involvement because, in contrast to traditional 2D imaging (such as panoramic radiographs), CBCT can provide a more comprehensive assessment of the actual extent of MRONJ [18,19,20]. Thus, only MRONJ cases examined with CBCT were included in this study.

CBCT is a valuable imaging technique in oral and maxillofacial surgery, and it is increasingly used to evaluate oral pathology even in the early stages of detection [21], with substantially reduced radiation exposure than multi-detector computed tomography (MDCT) [22].

In our study, the most common radiographic features were lytic changes. Osteolysis was observed in 82.7% of cases, agreeing with other MRONJ-related studies where osteolysis has generally been observed [8,9]. The second most common finding was sclerosis, which is considered an important bone alteration in patients with MRONJ [23,24]. In our study, sclerosis appeared in 75% of the affected jaws in all three clinical stages, a common finding in other studies [7,8,9].

Our results show that MRONJ presented a high occurrence of multiple or extensive bone sequestrations (53.8%) in all three clinical stages. These findings agree with other MRONJ-related studies that demonstrate the frequent presence of bone sequestration in this disease [8,25].

More than half of the cases in our study were classified into the CRIm medium group (4–8), consistent with the results of the other index-related studies [7,9].

We observed that in MRONJ clinical Stage 1, approximately half of the cases had low CRIm scores. In clinical Stage 2, the majority had medium CRIm scores, and in clinical Stage 3, most had high CRIm scores. This indicates the progression of the CRIm, as it tends to increase as the clinical stages advance, and this was found to be statistically significant. This indicates a correlation between MRONJ clinical and radiographic appearance, agreeing with other MRONJ-related studies [7,13,23].

A limitation of this study was the limited sample size. Further studies with more documented cases of MRONJ might contribute to evaluating and validating the effectiveness of the CRIm.

## 5. Conclusions

The CRIm appears to be a fairly valid indicator of MRONJ progression based on the extent of bone involvement. Moreover, the CRIm tends to increase with the advancement of MRONJ’s clinical stages, emphasizing the importance of including radiologic findings in the overall MRONJ patient assessment.

As imaging, especially CBCT, provides important information that may be missed by clinical examination alone, the CRIm could be a valuable tool for staging MRONJ severity.

To validate these findings, larger-sample studies are needed to assess the reproducibility and clinical applicability of the CRIm. Furthermore, investigating its integration with machine learning techniques for automated analysis could enhance its clinical utility by providing further insight into MRONJ disease progression and early diagnosis.

## Figures and Tables

**Table 1:** Localization of MRONJ findings in maxillae and mandibles divided into sextants.

Localization	(*n*, %)
Anterior maxilla	7 (13.5)
Posterior maxilla	11 (21.2)
Anterior mandible	18 (34.6)
Posterior mandible	33 (63.5)

**Table 2:** Correlation of the radiologic features with clinical stage of MRONJ.

Radiologic Features		Clinical Stage 1	Clinical Stage 2	Clinical Stage 3	*p*-Value
Lytic changes (*n*, %)	0	6 (31.6)	2 (12.5)	1 (5.9)	0.003 *
	1	8 (42.1)	3 (18.8)	1 (5.9)	
	2	5 (26.3)	11 (68.8)	15 (88.2)	
Sclerosis (*n*, %)	0	5 (26.3)	4 (25.0)	4 (23.5)	0.737
	1	2 (10.5)	2 (12.5)	0 (0.0)	
	2	12 (63.2)	10 (62.5)	13 (76.5)	
Periosteal bone formation (*n*, %)	0	13 (68.4)	10 (62.5)	8 (47.1)	0.499
	1	1 (5.3)	3 (18.8)	2 (11.8)	
	2	5 (26.3)	3 (18.8)	7 (41.2)	
Sequestration (*n*, %)	0	15 (78.9)	7 (43.8)	2 (11.8)	<0.001 *
	1	0 (0.0)	3 (18.8)	1 (5.9)	
	2	4 (21.1)	6 (37.5)	14 (82.4)	
Non-healing extraction sockets (*n*, %)	0	11 (57.9)	8 (50.0)	8 (47.1)	0.934
	1	5 (26.3)	4 (25.0)	6 (35.3)	
	2	3 (15.8)	4 (25.0)	3 (17.6)	
Other findings					
Sinus implication (*n*, %)	0	3 (60.0)	2 (66.7)	0 (0.0)	0.145
	1	2 (40.0)	1 (33.3)	5 (100.0)	
Inferior alveolar canal involvement (*n*, %)	0	14 (100.0)	13 (100.0)	2 (16.7)	<0.001 *
	1	0 (0.0)	0 (0.0)	10 (83.3)	
Jaw fracture (*n*, %)	0	19 (100.0)	16 (100.0)	15 (88.2)	0.193
	1	0 (0.0)	0 (0.0)	2 (11.8)	

Fisher’s exact test. * Statistically significant, α = 5%; 0: absent, 1: localized, 2: extensive. MRONJ—medication-related osteonecrosis of the jaw.

**Table 3:** Correlation between the clinical stage and the CRIm.

Clinical Staging	CRIm			
	Low (0–3)	Medium (4–8)	High (9–12)	*p*-Value
Clinical Stage 1	9 (47.4%)	9 (47.4%)	1 (5.3%)	0.010 ^1^*
Clinical Stage 2	3 (18.8%)	12 (75.0%)	1 (6.3%)	
Clinical Stage 3	2 (11.8%)	8 (47.1%)	7 (41.2%)	
	Rho = 0.446			0.001 ^2^*

1: Fisher’s exact test. 2: Spearman’s correlation coefficient. * Statistically significant, α = 5%.

## Data Availability

The datasets are available from the corresponding author upon reasonable request due to privacy/ethical restrictions.
